# Fas ligand expression in human and mouse cancer cell lines; a caveat on over-reliance on mRNA data

**DOI:** 10.1186/1477-3163-5-5

**Published:** 2006-02-02

**Authors:** Aideen E Ryan, Sinead Lane, Fergus Shanahan, Joe O'Connell, Aileen M Houston

**Affiliations:** 1 Department of Medicine, National University of Ireland Cork (NUIC), Clinical Science Building, Cork University Hospital, Wilton, Cork, Ireland; 2 Alimentary Pharmabiotic Centre, National University of Ireland Cork (NUIC), Ireland

## Abstract

**Background:**

During carcinogenesis, tumors develop multiple mechanisms for evading the immune response, including upregulation of Fas ligand (FasL/CD95L) expression. Expression of FasL may help to maintain tumor cells in a state of immune privilege by inducing apoptosis of anti-tumor immune effector cells. Recently this idea has been challenged by studies reporting that tumor cells of varying origin do not express FasL. In the present study, we aimed to comprehensively characterize FasL expression in tumors of both murine and human origin over a 72 hour time period.

**Methods:**

RNA and protein was extracted from six human (SW620, HT29, SW480, KM12SM, HCT116, Jurkat) and three mouse (CMT93, CT26, B16F10) cancer cell lines at regular time intervals over a 72 hour time period. FasL expression was detected at the mRNA level by RT-PCR, using intron spanning primers, and at the protein level by Western Blotting and immunofluorescence, using a polyclonal FasL- specific antibody.

**Results:**

Expression of FasL mRNA and protein was observed in all cell lines analysed. However, expression of FasL mRNA varied dramatically over time, with cells negative for FasL mRNA at many time points. In contrast, 8 of the 9 cell lines constitutively expressed FasL protein. Thus, cells can abundantly express FasL protein at times when FasL mRNA is absent.

**Conclusion:**

These findings demonstrate the importance of complete analysis of FasL expression by tumor cells in order to fully characterize its biological function and may help to resolve the discrepancies present in the literature regarding FasL expression and tumor immune privilege.

## Introduction

Recent advances in our understanding of the interactions of tumor cells with the immune system has resulted in the realisation that tumor cells devise multiple mechanisms to evade recognition by immune cells. Upregulation of Fas ligand (FasL/CD95L) expression may represent one such mechanism [1]. FasL functions to induce apoptotic cell death following ligation to its receptor Fas [2]. The Fas/FasL system plays an important role in immune homeostasis [3], and in the maintenance of immune privilege in sites such as the eye [4] and testis [5]. For instance, FasL expressed by non-lymphoid cells in the eye was shown to induce apoptosis of Fas^+ ^lymphoid cells entering in response to viral infection, thus protecting the eye from inflammatory damage. Following the discovery that tumor cells can express FasL, it was subsequently suggested that FasL may also confer immune suppression in malignancy and that this may represent a critical mode of tumor immune evasion – the 'Fas counterattack' [1,6,7].

However, this idea has recently been challenged [8]. Arguments raised against the 'Fas counterattack' include studies showing that ectopic overexpression of FasL in allografts of tissues or tumor cells leads in some cases to inflammation and rapid rejection [9,10]. Also, the actual ability of tumors to express FasL has been questioned, with reports suggesting that tumor cells of varying origin do not express FasL [11,12]. Furthermore, substantial concerns have been raised about the specificity of some commercially available anti-FasL antibodies [13].

In light of these concerns, the present study was designed to investigate the expression of FasL at both the mRNA and protein level, in cell lines of varying histological backgrounds (colon carcinoma, melanoma and lymphoma), and of both human and mouse origin. Expression of FasL mRNA and protein was observed in all cell lines analysed. However, while FasL expression varied over time at the mRNA level (+/-), FasL was consistently detected at the protein level throughout the culture period in eight of the nine cell lines analysed using two different FasL-specific antibodies. These results show that cells can abundantly express FasL protein at time points when FasL mRNA is absent and demonstrate the importance of complete analysis of FasL expression by tumor cells in order to fully characterize its biological function.

## Materials and methods

### Cells

SW620, HT29 and SW480 human colon adenocarcinoma cell lines and Jurkat T cell line were purchased from the American Type Culture Collection (ATCC) (Rockville, MD). The human colon carcinoma cell line, HCT116, was a gift from Prof. B. Vogelstein (The John Hopkins University School of Medicine, Baltimore, MD). The human metastatic colon carcinoma cell line, KM12SM, was kindly provided by Dr. Isaiah J Fidler (Department of Cancer Biology, University of Texas, MD Anderson Cancer Centre). B16F10, a mouse melanoma cell line, CT26 and CMT93, mouse colorectal carcinoma cell lines, were kindly donated by Dr. Stephen Todryk (Oxford University, UK).

Cells were grown in DMEM unless otherwise stated. KM12SM cells were grown in MEM supplemented with 2× vitamins, 1 mM sodium pyruvate and 1× non-essential amino acids. HCT116 were grown in McCoy's 5A. When analysing FasL expression, cells were grown in medium containing 100 U/ml penicillin, 100 μg/ml streptomycin, 0.5% fetal calf serum, 0.2% BSA, 10 μg/ml transferrin and 30 ηM sodium selenite.

### Analysis of FasL expression

Each cell line was seeded individually at ~2 × 10^5 ^cells /ml in 6 well plates. This was sufficient to achieve 20% confluency 18 hours later and this point was defined as 0 hour. FasL expression was monitored thereafter by reverse transcriptase-polymerase chain reaction (RT-PCR), Western blotting and immunofluorescence at regular intervals over the following 72 hours.

### RT-PCR detection of FasL mRNA transcripts

The expression of FasL mRNA was analyzed by RT-PCR. Total RNA was isolated using Tri Reagent (Molecular Research Centre, Cincinnati, OH), according to the manufacturer's instructions. 1.5 μg of total RNA was used for first strand cDNA synthesis in a mixture containing AMV Reverse Transcriptase, random hexanucleotide primers, RNasin (40 U) and dNTPs (500 μM) (Promega, WI). Subsequently, FasL cDNA was amplified by 35–40 cycles of PCR using the following intron-spanning primers: human FasL – forward 5'- GGATTGGGCCTGGGGATGTTTCA- 3' and reverse 5'-TTGTGGCTCAGGGGCAGGTTGTTG-3' (344 bp product); mouse FasL – forward 5'-CGGTGGTATTTTTCATGGTTCTGG-3' and reverse 5'-CTTGTGGTTTAGGGGCTGGTTGTT-3' (380 bp). To ensure comparable amplification efficiencies in all RNA samples, β-actin RT-PCR was also performed using the following primers: forward 5'-CCTTCCTGGGCATGGAGTCCTG-3' and reverse 5'-GGAGCAATGATCTTGATCTT C-3' (202 bp). PCR products were resolved on a 2% agarose gel and visualized by ethidium bromide staining.

### Western blotting detection of FasL protein

Cells were rinsed once in PBS and lysed on ice for 5 mins in 20 mM Tris-HCl, pH 7.4, containing 150 mM NaCl and 1% triton × 100, supplemented with the complete-TM mixture of protease inhibitors (Roche Molecular Biochemicals, Indianapolis, IN). Lysates were sonicated and cleared by micro centrifugation at 10,000 g for 5 mins. Protein concentrations were determined using the BCA protein assay (Pierce Chemicals, Rockford, IL). Equal amounts of protein (50 μg for FasL; 15 μg for β-actin) were separated in a 10% SDS polyacrylamide gel and transferred to a nitrocellulose membrane. Protein loading and efficiency of transfer were monitored by Ponceau S staining. Membranes were blocked for 1 hour at room temperature in Blotto (5% non-fat dry milk in 0.1% PBS/Tween-20) and then incubated overnight at 4°C with 1 μg/ml rabbit polyclonal anti-FasL antibody (Ab-1; Oncogene, CA), 0.1 μg/ml rabbit polyclonal anti-FasL antibody (N-20; Santa Cruz, CA) or mouse monoclonal anti-β-actin specific antibody (1:10,000) (Clone AC-74; Sigma, UK). Primary antibodies were detected with horseradish peroxidase-conjugated IgG (Dako Corp., Carpinteria, CA) raised against the corresponding species followed by ECL visualization (Pierce Chemicals).

### Immunofluorescent detection of FasL in human and mouse cell lines

For immunostaining, slides were fixed with 1.5% paraformaldehyde and then blocked with 10 mg/ml BSA plus 1% rabbit serum for 30 minutes at room temperature. Human cell monolayers were incubated overnight at 4°C with 2 μg/ml anti-FasL specific antibody (G247-4; Pharmingen, UK) while mouse cell monolayers were incubated with 20 μg/ml of monoclonal anti-FasL specific antibody (A11; Alexis Biochemical, UK) for 1 hour at room temperature. Antibody binding was localized using a FITC-labeled secondary antibody (Dako). Slides were mounted with anti-fading media (Dako) and visualized using a fluorescent microscope when dry.

## Results

### Cancer cell lines of varying origin express FasL mRNA and protein in a cyclic manner

**Figure 1:**
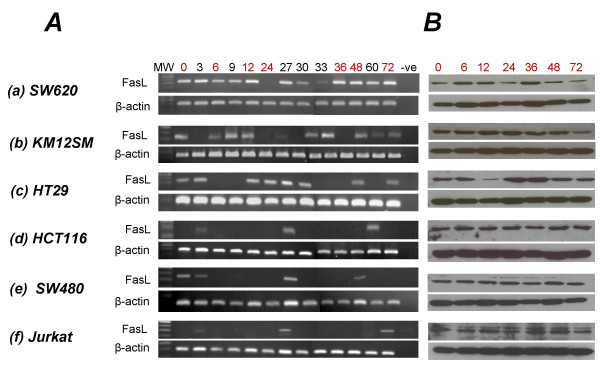
**Characterisation of FasL expression in tumor cell lines of human origin**. **A**: FasL mRNA expression by each cell line was analyzed at the indicated times by RT-PCR. β-actin RT-PCR was performed to monitor RT-PCR amplification efficiency, with all samples yielding equivalent levels. **B**: FasL protein expression was monitored by immunoblot analysis using the Ab-1 anti-FasL antibody (Oncogene). Results are representative of three independent experiments.

Given the recent discrepancies in the literature regarding FasL expression by various tumor cell lines, FasL mRNA expression by six human tumor cell lines (one T-cell lymphoma and five colon carcinomas) was analysed by RT-PCR. Expression was examined at three hour intervals over a period of 72 hours. Intron spanning primers were used to avoid amplification of contaminating genomic DNA. The FasL mRNA transcript was detectable at numerous time points throughout the 72 hour culture period (Figure 1A). All cell lines investigated expressed FasL mRNA; however, expression varied dramatically over time, with cells negative for FasL mRNA at many time points. Despite this cyclic pattern of expression, coupled with very low levels of FasL mRNA for some cell lines (e.g. HCT116 colon carcinoma cells were positive for FasL mRNA at only 3 of the 13 time points examined (3, 27 and 60 hours)), all cells were observed to constitutively express FasL protein as assessed by Western blotting (Figure 1B).

**Figure 2:**
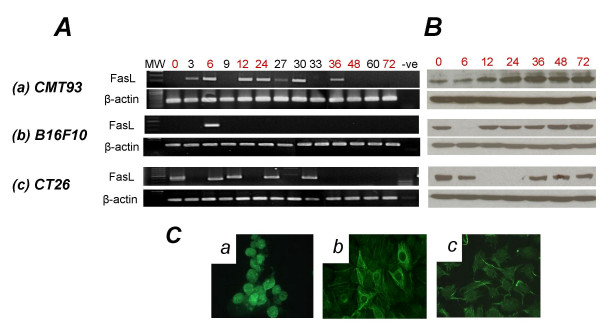
**Analysis of FasL expression in murine tumor cell lines**. **A**. FasL expression by murine tumor cells was analyzed by RT-PCR at the indicated times. **B**. FasL protein expression was monitored by Western blotting using the Ab-1 anti-FasL antibody. **C**. After 48 hrs culture, FasL expression was detected by immunofluorescence on paraformaldehyde-fixed cell monolayers. Representative cell lines are shown – (a) EL-4 (murine T-cell line – positive control), (b) CMT93 and (c) B16F10. Results are representative of three independent experiments.

To determine whether this phenomenon was conserved across different species, FasL expression was also analysed in three tumor cell lines of murine origin (two colon carcinoma and one melanoma) (Figure 2A). Again expression varied dramatically over time. In fact, B16F10 melanoma cells expressed FasL mRNA at just a single time point (6 hours). Despite this, Western blot analysis revealed constitutive FasL protein expression (Figure 2B). In contrast, CT26 colon carcinoma cells expressed FasL mRNA at 5 of the 13 time points, yet FasL protein was not detected following 12 and 24 hours in culture.

Additional confirmation of the lack of correlation between FasL mRNA and protein expression was provided by immunofluorescent staining (Figure 2C). At 48 hours, B16F10 and CMT93 cells expressed comparable levels of FasL protein, yet both of these cell lines fail to express FasL mRNA at this time. Moreover, CMT93 cells overall express substantially higher levels of FasL mRNA than B16F10 cells.

### Confirmation of antibody specificity

**Figure 3:**
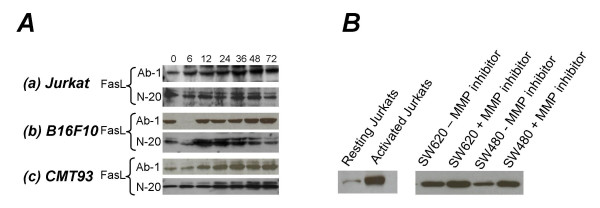
**Confirmation of the specificity of FasL protein detection**. **A**. FasL protein was detected by Western blotting using both the Ab-1 and N-20 anti-FasL antibodies. Results from three representative cell lines are shown. **B**. FasL protein production was also detected by Western blotting using the Ab-1 antibody following PHA activation of Jurkat cells and treatment of SW620 and SW480 cells with MMP inhibitors. Results are representative of three independent experiments.

Another argument levelled against the 'Fas counterattack' as a mechanism of tumor immune evasion relates to the specificity of some commercially available antibodies. For this reason, in our study we used two different non-contested antibodies (Ab-I, Oncogene; N-20, Santa Cruz) for Western blotting. Both antibodies demonstrated a similar pattern of FasL expression (Figure 3A). The specificity of the antibodies was further validated by activating Jurkat T cells using phytohemagglutinin (PHA). *In vitro*, T cell activation leads to increased expression of FasL. PHA-activated Jurkat T cells exhibited substantially increased levels of FasL protein (Figure 3B). Also, increased expression of FasL was detected in SW620 and SW480 cells following treatment with matrix metalloproteinase (MMP) inhibitors (Figure 3B). MMP inhibitors prevent the cleavage of FasL from the surface of the cells, leading to increased levels of membrane-bound FasL.

### The cyclic nature of FasL expression is not due to the degree of confluency of the cells or cellular stress *in vitro*

**Figure 4:**
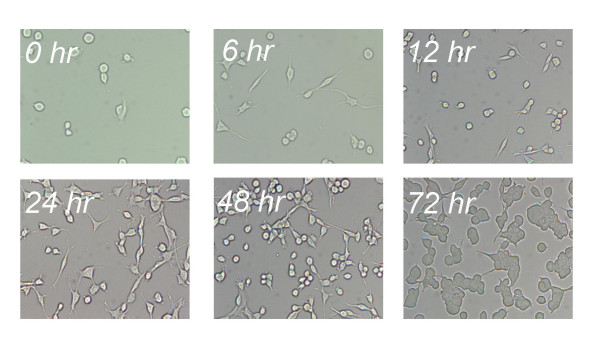
**CT26 cell morphology during the course of FasL analysis**. Cell lines were seeded at ~2 × 10^5 ^cells/ml in 6 well plates to achieve 20% confluency 18 hours later. This point was defined as 0 hr. After 72 hrs, cells had reached ~80–90% confluency.

Several parameters may regulate the expression of FasL *in vitro*, including confluency, serum deprivation, growth factor withdrawal and cellular stress [14,15]. To minimise the effect of these factors, all tumor-derived cell lines were grown in serum-supplemented media containing essential nutrients. Cells were plated at ~20% confluency and after 72 hours in culture were viable and exhibited ~80–90% confluency (Figure 4). Consequently, the cyclic nature of FasL expression observed in this report was not due either to the cells becoming over-confluent or to cellular stress. In fact, six of the nine cell lines exhibited reduced or negligible levels of FasL mRNA at 72 hours.

## Discussion

The role of FasL in tumor immune evasion and immune privilege is controversial [8,16]. Arguments leveled against a role for FasL include the failure of some investigators to detect FasL expression by tumor cells, together with the lack of specificity exhibited by some commercially available antibodies. In the present study, we characterized FasL expression by nine different tumor cell lines of varying origin. We found that cells can maintain constitutive FasL protein expression, despite extensive variations in, and at times absent, FasL mRNA production.

These findings provide a rational explanation for some of the conflicting reports present in the literature regarding FasL expression. For instance, Rudi and colleagues demonstrated the presence of FasL mRNA in 2 gastric carcinoma cell lines, including Kato III cells [17]. However, Tinhofer *et al. *were unable to detect FasL mRNA in this cell line [12]. Also, Hahne *et al. *reported that melanoma cells, including B16F10 cells [18], express FasL, while Chappell *et al. *were unable to detect FasL mRNA in a panel of early passage melanoma cell lines [11]. In all of these reports, FasL mRNA expression was analysed at a single time. The results of the current study clearly show that analysis at a single time point is insufficient to conclude the presence or absence of FasL expression. Depending on the time at which the cells are analysed, tumors may or may not express FasL mRNA. These findings caution against relying on a single time point when analysing the expression of mRNA, and emphasize the importance of confirming any findings at the protein level.

Much of the controversy surrounding the 'Fas counterattack' as a mechanism of tumor immune evasion stems from transplantation studies. As a mediator of immune privilege, it was hoped that FasL could be used to prevent rejection of allografts. However, forced, overexpression of FasL in allografts or tumor cells induced accelerated destruction in many instances via recruitment of neutrophils [9,10,19]. Many rodent transplantation studies have utilised CT26 colon tumor cells, which they reported to be negative for FasL expression [9,19]. However, extensive characterisation of this cell line in the current study revealed that these cells do express FasL, albeit in a cyclic fashion, at both the mRNA and protein level. Thus, transfection of these FasL-expressing cells with recombinant FasL may result in an excessive level of expression of FasL by these cells. Indeed, downregulation of FasL expression was recently shown to result in an increased anti-tumor immune challenge and decreased tumor formation *in vivo*, providing functional evidence in favor of the 'Fas counterattack' as a mechanism of tumour immune evasion [20]. The level of FasL expression has been shown to be important in mediating immune privilege [21,22], with excessive levels of FasL being linked to inflammation, perhaps due to excessive apoptosis in the vicinity of the allograft [23]. However, selective pressure during the gradual evolution of a spontaneous tumor would ensure that FasL upregulation could only occur at a level where it would be advantageous to the tumor. Furthermore, to date, naturally expressed FasL has not been shown to possess a potent pro-inflammatory activity and is instead associated with immunosuppressive and anti-inflammatory effects.

Recent publications have suggested that pre-formed FasL can be stored in microvesicles, which can traffic to the cell surface and induce apoptosis of Fas-bearing sensitive cells [24,25]. Thus, the ability of FasL-expressing cells to trigger apoptosis may not depend on *de novo *protein synthesis. Instead, FasL protein may be present in a cell, in the absence of FasL mRNA. These findings are consistent with those of the current study and suggest that FasL may be regulated at the transcriptional level. Following the synthesis of sufficient amounts of FasL protein, the level of FasL mRNA may decline, perhaps to prevent the translation of excessive amounts of FasL protein which could potentially trigger neutrophil recruitment. Cyclic variation in the expression of FasL was seen in cells of both mouse and human origin, suggesting that this represents an important means of regulating FasL production for cells.

## Abbreviations

FasL, Fas ligand; RT-PCR, reverse transcriptase-polymerase chain reaction; PHA, phytohemagglutinin; MMP, matrix metalloproteinase.

## Authors' contributions

AR performed the RT-PCR, Western blotting and immunofluorescence, and the experiments confirming the antibody specificity. AH and SL participated in the RT-PCR and Western blotting on the human cell lines. FS, JOC and AH participated in the study design and co-ordination. AH and AR drafted the manuscript.

All authors read and approved the final manuscript.
